# Handling the challenges of accessing reliable and tailored information on physical activity during pregnancy: a mixed-methods, cross-perspective investigation

**DOI:** 10.1186/s12884-026-09482-7

**Published:** 2026-06-15

**Authors:** Mathilde Hyvärinen, Mireille van Poppel, Hélène Legardeur, Maxime Haubry, Jennifer Wegrzyk, Claire de Labrusse

**Affiliations:** 1 HESAV School of Health Sciences - Vaud, HES-SO University of Applied Sciences and Arts Western Switzerland, Avenue de Beaumont 21, Lausanne, 1011 Switzerland; 2 Department of Human Movement Science, Sport and Health, University of Graz, Mozartgasse 14, Graz, 8010 Austria; 3 Department Woman-Mother-Child, Lausanne University Hospital (CHUV) and University of Lausanne, Avenue Pierre-Decker 2, 1011 Lausanne, Switzerland; 4 Department Woman-Mother-Child, Neuchâtel hospital network (RHNe), Rue de la Maladière 45, 2000 Neuchâtel, Switzerland

**Keywords:** Physical activity, Pregnancy, Behavior change theories, Maternity care, Health promotion

## Abstract

**Background:**

Physical inactivity during pregnancy remains a major public health challenge. Although many interventions have been shown to significantly increase physical activity (PA), their real‑world impact remains limited. Healthcare professionals represent a key lever for addressing this challenge, yet PA is still insufficiently promoted despite recognized health benefits. Embedding PA promotion into maternity care, particularly through midwives, appears promising, but how to ensure equitable implementation that supports informed decision‑making in practice remains unclear. This study therefore aims to better understand how behavioral determinants, professional PA promotion practices, and pregnant individuals’ characteristics collectively influence decisions to engage in PA during pregnancy and to inform a framework for implementation.

**Methods:**

A mixed‑methods study was conducted in two maternity hospitals in Western Switzerland. Ninety‑five pregnant women completed a survey on their perceptions of PA communication across professions, and 16 participated in semi‑structured interviews. Additionally, 19 hospital‑based midwives were interviewed about their practices. Data collected in parallel were analysed separately using descriptive and inferential statistics, and thematic analysis informed by the Theoretical Domains Framework (TDF) and the Capability‑Opportunity‑Motivation‑Behavior (COM‑B) model. Integration of findings occurred after separate analyses using the Pillar Integration Process.

**Results:**

Most participants were in their third trimester (76%), highly educated (79%), and followed by private obstetrician‑gynecologists (82%). Access to PA information often relied on women’s own proactivity, with 37% receiving no information and 59% wanting more. Incomplete and non-spontaneous communication across professions negatively shaped women’s PA decision‑making due to misbeliefs and negative emotions. Hospital‑based midwives frequently forgot or avoided the topic, especially when unconfident or perceiving low interest, due to limited training, procedures, and resources, contributing to inconsistent and inaccurate PA communication. Together, these insights informed a COM‑B‑based framework designed to strengthen hospital-based midwives’ capability, opportunity, and motivation and promote spontaneous PA communication targeting knowledge, beliefs about consequences, and emotion to support tailored decision about PA during pregnancy.

**Conclusion:**

This study provides insights into how multilevel determinants shape access to PA information. It presents a theory‑driven framework to guide strategies for integrating systematic, spontaneous, and tailored PA communication into care to support informed decisions to engage in PA during pregnancy.

**Supplementary Information:**

The online version contains supplementary material available at 10.1186/s12884-026-09482-7.

## Background

 The constant rising prevalence of non-communicable diseases, overweight, and obesity worldwide presents a complex public health challenge that could be mitigated by physical activity (PA) [1]. PA during pregnancy is associated with a reduced risk of several pregnancy-related complications, including gestational diabetes, preeclampsia, depression, and macrosomia, and contributes to long-term health [2]. During this period, frequent interactions with healthcare professionals offer a unique opportunity to effectively communicate on PA recommendations to every family. This stage of life represents a “window of opportunity”, as parents are generally more receptive to health information, including PA benefits, which can result in lifelong benefits for the entire family [3, 4].

According to international pregnancy-specific PA guidelines, pregnant individuals with no contraindication should accumulate at least 150 min of moderate-intensity aerobic PA per week, complemented by muscle-strengthening activities, mobility and pelvic floor exercises, while also reducing sedentary time [1]. Those already active can continue moderate-to-vigorous-intensity activities, and those less active should increase PA gradually [1].

Professional guidelines therefore clearly state that PA during this period should be addressed as early as possible in a tailored and motivational way [5]. Healthcare professionals, particularly midwives, are recognized as trusted sources of information and key influencers of PA behavior during the transition to parenthood [5–8]. However, despite their recognized role, midwives often do not promote accurately PA during pregnancy [9–11], while physical inactivity remains a significant public health concern contributing to adverse maternal and child health outcomes [5, 12].

Physical inactivity during pregnancy results from multiple interacting individual, socioeconomic, and environmental determinants, including knowledge, skills, time constraints, physical discomfort, income, education, guidance from healthcare professionals, cost, weather, and societal perceptions of prenatal PA [13–19]. Although interventions targeting both overweight and healthy pregnant women have demonstrated positive effects on PA levels, well-being, and health [20–22], their integration into routine care remains limited, and many still do not receive adequate information on PA [8, 10, 23–25]. Healthcare professionals, including midwives, face significant barriers such as lack of time, knowledge, skills, and confidence, as well as system‑level constraints including insufficient training, lack of reimbursement of PA counselling, unclear procedures and role, heavy workloads, and low institutional prioritization of PA promotion [10, 19, 25–29]. To address these challenges, identified strategies include enhancing initial and ongoing training and providing resources as time, leaflets, mobile applications and prenatal PA classes [10, 27, 30–33]. These strategies should ensure the delivery of consistent, accurate and tailored information to support informed decision‑making regarding PA during pregnancy [14, 19, 34].

Although the safety and benefits of PA are well established [2], and the individual-level determinants influencing both prenatal PA behaviors and professional PA promotion practices are well described, little is known about how to implement PA promotion strategies sustainably in real-world care. Most existing studies have investigated determinants using qualitative approaches with limited use of multilevel operational frameworks and have assessed PA promotion strategies under controlled conditions, with limited research on their implementation under real-world care conditions [7, 8, 19, 35].

Behavior change theories provide valuable frameworks for understanding the determinants of complex health behaviors and related them to effective strategies [36, 37], and have already been used in the context of pregnancy [4, 7]. However, existing studies have largely focused either on professional PA promotion practices or on prenatal PA behaviors, with limited examination of how these determinants interact within real-world care and how strategies based on theoretical frameworks can be implemented in practice [10, 22, 27, 38–40].

Consequently, how multilevel determinants shape PA communication within care relationships and influence PA engagement during pregnancy in real-world care settings remains insufficiently understood.

This mixed-methods study therefore aims to better understand how behavioral determinants, professional PA promotion practices, and pregnant individuals’ characteristics collectively influence decisions to engage in PA during pregnancy. Building on this understanding, this article also develops a framework to guide effective strategies for integrating PA promotion into routine care through midwives, with a focus on communication and access of reliable information.

## Methods

### Participants and recruitment

#### Settings

The study was conducted at the maternity department of the Lausanne University Hospital (CHUV), and at the maternity department of Neuchâtel hospital network (RHNe) in Western Switzerland. These sites were selected to increase participant diversity: a large university hospital and a regional hospital.

In Switzerland, maternity care is provided either in maternity hospitals or by independent obstetrician-gynecologists, with final consultations taking place in hospital before birth. Pregnant individuals typically interact with both midwives and obstetrician-gynecologists during pregnancy and postpartum.

#### Recruitment

A purposive sampling strategy was used to recruit pregnant individuals and midwives in 2022 at CHUV and in 2023–2024 at RHNe. No prior relationship existed between the researchers and participants. Pregnant participants were eligible if they were ≥ 18 years old, had at least B1 proficiency in French, German, or English, had no absolute contraindication to PA, and were currently pregnant or had given birth within the past month, whether they were followed in a hospital setting or by independent healthcare professionals. Reporting from participants with placenta previa were retained as the survey collected their perceptions in the first and second trimesters. Hospital-based midwives were eligible if involved in prenatal consultations or childbirth/parenthood preparation classes.

Pregnant participants were recruited through posters, flyers, and in-person invitations by the first author (MHy) during her regular presence in waiting rooms. Participants could complete a survey, participate in an interview, or both. Of the 174 scans providing access to the online consent form, 95 surveys contained data on perceived professional practice regarding PA. Among the 38 women who provided contact details, 16 participated in interviews.

All eligible midwives were invited during a presentation and by e-mail. Approximately half of midwives participated based on availability and personal interest, resulting in varied years of experience and profiles. Interviews were performed during working hours.

Participants were informed about the study objectives, including improving PA promotion during pregnancy care. Pregnant participants provided online consent before completing the survey or sharing contact details; oral consent was reconfirmed at the start of interview. Midwives provided first written consent with contact details and reconfirmed orally before interview. Up to three e‑mail invitations were send to all potential interviewees. No incentives were offered, and participants could withdraw at any time.

### Theoretical framework

A recognized theoretical framework was used to support a comprehensive understanding of the targeted behaviors and inform the development of a future intervention to promote PA during pregnancy in routine care. The Theoretical Domains Framework (TDF) was used to guide the identification of behavioral determinants [41], in alignment with the Capability–Opportunity–Motivation–Behavior (COM-B) model [37].

The TDF synthesizes 33 behavior change theories into 14 domains describing determinants of behavior [41]. The COM-B model groups these domains into three core constructs, Capability, Opportunity, and Motivation, within the Behavior Change Wheel [37]. These constructs are linked to intervention functions and behavior change techniques designed to support behavior change [37, 42].

### Design and procedures

A descriptive convergent mixed‑methods design was used to examine PA promotion practices from the complementary perspectives of pregnant women and hospital‑based midwives. This approach, well suited to healthcare research, combines quantitative and qualitative data to capture the complexity of real‑world clinical care [43]. Quantitative (QUAN) data offered an initial overview of prenatal PA promotion practices in Switzerland, while qualitative (QUAL) data explored the mechanisms shaping communication about PA during pregnancy. Together, these components enabled the analysis of multilevel determinants within routine care.

Quantitative (survey) and qualitative (interview) data were collected concurrently, analyzed separately, and then integrated [43]. Both components received equal weighting to investigate how professional practices relate to women’s decision‑making [44]. Integration followed the Pillar Integration Process (PIP) [45], which involves listing, matching, checking, and pillar‑building to merge datasets and generate meta‑inferences through structured joint displays [46].

#### Survey (QUAN)

Pregnant participants completed an anonymous self-administered online survey accessed via QR code. The first section used a validated instrument to assess PA level [47], while the second section included study-specific items on PA promotion practices (see Additional file 1). Only the second section is analyzed in the present study.

The completion time for the second part was estimated at 5 min, whereas the first part required about 10 min. Items assessed sociodemographic characteristics (age group, education, nationality, pregnancy trimester, type of follow-up), perceived pre-pregnancy PA level, and perceived knowledge of PA recommendations. Participants were asked whether they had received information about PA at least once from any healthcare professional (e.g., obstetrician-gynecologist, midwife, nurse, physiotherapist, etc.). They were also asked whether the advice was to reduce, maintain, or increase PA, and whether they were satisfied with the information received. Items were aligned with interview guides to support data integration.

#### Interview (QUAL)

Midwives and pregnant women were recruited until no substantially new insights were identified. For pregnant women, recruitment also ceased because remaining eligible profiles declined participation despite repeated invitations.

Interviews lasted approximately 30–45 min and were conducted in person or online according to participant preference. Interview guides were developed from relevant literature [10, 14, 27] and team expertise [10, 48], available in Additional file 1.

All interviews were conducted by the first author (MHy), a female specialist in adapted physical activity (MSc Sport Science, PhD candidate) with prior experience in PA during pregnancy, trained in motivational interviewing, and mentored. Interviews were audio-recorded, transcribed verbatim, coded and accompanied by field notes documenting contextual information and preliminary analytic insights.

### Thematic and statistical analysis

#### Survey (QUAN)

Data were analyzed using descriptive and inferential statistics in R version 4.3.1. Percentages were calculated based on the number of respondents per item due to optional questions and conditional display.

Associations between categorical variables were examined using Chi-square tests after verifying expected cell frequencies. Responses to multiple-choice questions were grouped into two or three categories. Non-substantive responses (e.g., “prefer not to answer”, “don’t know”) were treated as missing data. Cases with missing values were excluded from inferential analyses when required to meet statistical assumptions.

#### Interview (QUAL)

A Framework Method analysis was conducted as described by Gale et al. (2013), with TDF domains as framework [36, 49]. Data were managed in ATLAS.ti 8.

Analysis involved familiarization with transcripts, deductive coding using TDF domains with inductive sub-codes, development of an analytical framework, and charting data into a TDF framework matrix mapped into COM-B components. Patterns across domains were interpreted to generate themes describing behavioral determinants and mechanisms.

Two researchers (MHy and CdL) double-coded 10% of transcripts to ensure consistency, with the remaining transcripts coded by MHy and discussed through peer debriefing. Themes were developed separately for midwives and pregnant women. Only themes directly relevant to professional practices and women’s decisions to engage in PA were retained (see Additional file 2).

#### Integration (QUAN + QUAL)

Integration occurred at the interpretation stage following separate analyses. Using the PIP, QUAN and QUAL findings were first listed independently, then matched to identify convergence, divergence, and complementarity [45]. Matches were cross-checked against the original datasets, and integrated “pillars” were generated to develop overarching themes and meta-inferences [45]. Pillars synthesize datasets by bringing women’s QUAN statistics and QUAL experiences, mainly shaped by independent obstetric care, together with hospital‑based midwives’ QUAL insights. This integration offers illustrative and comparable insights from both perspectives, strengthening the completeness of the interpretation. Additional files 3 and 4 illustrate the final pillar-building process.

### Ethical issues

Ethical review and approval were waived, as the Human Research Ethics Committee of Canton Vaud (CER-VD; Req-2022-00094) confirmed that the study falls outside the scope of the Swiss Federal Act on Research Involving Humans.

## Results

### Quantitative results – women’s perspective

#### Pregnant participant characteristics

The socio-demographic characteristics of the 95 pregnant women are presented in Table 1. Of the participants, 72 (76%) were recruited at CHUV and 23 (24%) at RHNe. Participants were mostly in the third trimester of pregnancy (76%), reported a high education level (79%), and mainly received prenatal care from private independent obstetrician-gynecologists (82%). Approximately 62% reported pregnancy-related conditions, mainly joint pain (37%), water retention or varicose veins (25%), anemia (19%), gestational diabetes (5%), excessive weight gain (4%), and placenta previa (3%).

**Table 1:** Socio-demographic characteristics and self-perceived physical activity level and knowledge among pregnant women in Switzerland

Variable	CHUV (*n* = 72) *n* (%)	RHNe (*n* = 23) *n* (%)	Total (*n* = 95) *n* (%)
Age (years)			
18–24	0 (0.0)	2 (8.7)	2 (2.1)
25–30	14 (19.4)	4 (17.4)	18 (18.9)
31–40	56 (77.8)	17 (73.9)	73 (76.8)
40+	2 (2.8)	0 (0.0)	2 (2.1)
Level of Education			
Compulsory school	2 (2.8)	1 (4.3)	3 (3.2)
Secondary level	11 (15.3)	3 (13.0)	14 (14.7)
Tertiary level	58 (80.6)	17 (73.9)	75 (78.9)
Do not wish to reply	1 (1.4)	2 (8.7)	3 (3.2)
Nationality			
Swiss	40 (55.6)	15 (65.2)	55 (57.9)
Other	32 (44.4)	8 (34.8)	40 (42.1)
Gestation			
First trimester	3 (4.2)	0 (0.0)	3 (3.2)
Second trimester	16 (22.2)	3 (13.0)	19 (20.0)
Third trimester	52 (72.2)	20 (87.0)	72 (75.8)
Do not know	1 (1.4)	0 (0.0)	1 (1.1)
Main place of care			
Private independent obstetrician-gynecologist	56 (77.8)	22 (95.7)	78 (82.1)
Private independent midwife	1 (1.4)	0 (0.0)	1 (1.1)
Maternity hospital	15 (20.8)	1 (4.3)	16 (16.8)
Perceived pre-pregnancy level of PA			
Inactive (≤ 30 min/week of moderate intensity)	4 (5.6)	1 (4.3)	5 (5.3)
Partially active (30–149 min/week of moderate intensity)	26 (36.1)	9 (39.1)	35 (36.8)
Active (≥ 150 min/week of moderate intensity)	24 (33.3)	7 (30.4)	31 (32.6)
Very active (performing vigorous intensity regularly)	18 (25.0)	6 (26.1)	24 (25.3)
Perceived knowledge of PA recommendations during pregnancy			
Yes	13 (18.1)	6 (26.1)	19 (20.0)
Rather yes	39 (54.2)	12 (52.2)	51 (53.7)
Rather no	14 (19.4)	3 (13.0)	17 (17.9)
No	6 (8.3)	1 (4.3)	7 (7.4)
No opinion	0 (0.0)	1 (4.3)	1 (1.1)

*PA* Physical activity, *CHUV* Lausanne University Hospital, ~ 3,000 births/year, *RHNe* Neuchâtel Hospital Network, ~ 1,000 births/year

The participant distribution roughly reflects the annual birth ratio between institutions

#### Pre-pregnancy physical activity level and knowledge

Self-perceived pre-pregnancy PA level and knowledge of PA recommendations during pregnancy are shown in Table 1. Approximately 40% of women reported not meeting PA recommendations before pregnancy (inactive and partially active), and one quarter considered themselves very active. Regarding knowledge of recommendations, about one quarter reported limited or no knowledge, half reported some knowledge, and one fifth reported complete knowledge.

Women with higher pre-pregnancy PA levels (active and very active) were more likely to report knowing or partially knowing PA recommendations compared with those with lower PA levels (inactive and partially active) (χ² (1) = 4.2, *p* = 0.04).

#### Perception of professional practice

Perceptions of professional practices are presented in Table 2. Slightly less than 40% of participants reported not receiving any information about PA during pregnancy. When PA was addressed, healthcare professionals most often recommended maintaining current PA habits. No one recommended increasing them according to participants. The participants who reported placenta previa were advised to maintain their PA.

Among women reporting pregnancy-related conditions, about half received PA information related to their condition (*n* = 27, 51%). Fewer than half of participants were satisfied or very satisfied with the (non-)information received from healthcare professionals. Many women expressed a desire for more information (59%). Women who discussed PA with a healthcare professional were more likely to report wanting more information (χ² (2) = 13.3, *p* = 0.001).

**Table 2:** Perception of physical activity promotion practices reported by pregnant women in Western Switzerland

Variables	CHUV (*n* = 72) *n* (%)	RHNe (*n* = 23) *n* (%)	Total (*n* = 95) *n* (%)
Type of guidance received on PA			
None	28 (38.9)	7 (30.4)	35 (36.8)
To reduce	7 (9.7)	3 (13.0)	10 (10.5)
To maintain	33 (45.8)	10 (43.5)	43 (45.3)
To increase	0 (0.0)	0 (0.0)	0 (0.0)
Other	3 (4.2)	1 (4.3)	4 (4.2)
Missing data	1 (1.4)	2 (8.7)	3 (3.2)
Participants satisfied with information on PA	32 (44.4)	11 (47.8)	43 (45.3)
Did not receive information	3 (4.2)	0 (0.0)	3 (3.2)
Received information	29 (40.3)	10 (43.5)	39 (41.1)
Participants wished to receive more information on PA	43 (59.7)	13 (56.5)	56 (58.9)
Did not receive information	18 (25.0)	5 (21.7)	23 (24.2)
Received information	24 (33.3)	8 (34.8)	32 (33.7)

Responses on Likert scales were grouped as 1–2 for unsatisfied or wanted no more information, 3 as neutral, and 4–5 for satisfied or wanted more information. Satisfaction was assessed regardless of whether information was reported as received

*PA*  Physical activity, *CHUV*  Lausanne University Hospital, *RHNe*  Neuchâtel Hospital Network

### Qualitative results – women’s perspective

#### Pregnant participant characteristics

Sixteen women participated in semi-structured interviews. Eleven were recruited at CHUV and five at RHNe. Most reported participating because they perceived a lack of information on the topic and hoped the situation would improve.

Seven were pregnant with their first child and five with their second. Three women were in the second trimester and nine in the third trimester. In addition, four women had given birth within the previous month, two to their first child and two to their second.

All participants reported being at least partially physically active before pregnancy. Most of them practiced PA as leisure time. During pregnancy, two women reported being inactive, while most remained active but at a lower level than pre-pregnancy. All participants had tertiary education.

#### Determinants influencing decisions about physical activity engagement during pregnancy

Tables 3 and 4 present the summary matrix of determinants with illustrative quotes. The narrative interpretation of analytic themes is presented below, with the full set of themes and sub‑themes provided in Additional file 3. 

**Table 3:** How women’s characteristics shape care interactions and access to information about physical activity during pregnancy

Behavioral Determinants* (TDF-COM-B)	Barriers	Examples of quote	Summary
Social Influences - Social Opportunity	- Social Influences of HCP were mostly cited as a barrier associated with lack of knowledge. - The absence of information or the incomplete information from HCP negatively influenced the decision-making process.	*The main difference I saw was that in other areas it was very clear: “Yes*,* eat that. No*,* don’t eat that”. And the gynecologist had a clear opinion*,* so it was easy to use. But when it came to physical activity*,* there was no clear recommendation. It was never precise. They just didn’t really seem to know and let you try it.* (Pregnant women 5, nulliparous, third trimester, independent obstetrician) *I think that*,* depending on the importance of the question*,* it’s up to them to say so. [without I had to ask].* (Pregnant woman 8, nulliparous, third trimester, independent obstetrician)	Most women perceived the lack of knowledge of HCP about PA and deplored the conflicting and incomplete discourse of various HCP. They reported that PA topic was only once shortly and vaguely addressed at the beginning of pregnancy if they asked question about it. They would have liked to receive information adapted to their profile and pregnancy-related conditions all over the course of pregnancy. They wished that HCP investigate more their PA practices and sedentary behavior, and address PA in a clear, precise, and systematic way at several consultations, like other health behaviors.
Environmental Context and Resources - Physical Opportunity	- Lack of reliable Environmental Context and Resources were cited as a barrier and associated with the lack of Knowledge. - Reliable informative Resources when found influenced positively the decision-making process.	*I got several types of messages. From the obstetrician*,* very little. She wasn’t very interested in getting into the subject. I went to see an osteopath. She told me: “But you know*,* Madam*,* the only thing that’s recommended is walking and swimming”. [.] It’s true that I didn’t ask very precise questions about what type of movement. I had the impression that I’d got it from my reading or from the coaches in the United States.* (Pregnant women 5, nulliparous, third trimester, independent obstetrician) *I’ve been told that it’s important not to stay in bed*,* that you must keep moving. But that’s all. It’s more me really. I nearly had gestational diabetes. And then I said to myself: I’m going out*,* I’m not eating sugar. But it was more me than my obstetrician.* (Post-partum women 8, two children, independent obstetrician) *I think that since we received a flyer on nutrition from the WHO with the recommendations on whether you should eat this*,* you shouldn’t eat that. Well*,* I think that just a little sheet of information like that for such and such trimester or such and such month*,* that’s recommended*,* that’s to be avoided. It would have given me a line to follow.* (Pregnant woman 8, nulliparous, third trimester, independent obstetrician)	The vast majority reported not being satisfied with the answers they received and looked for information on their own. Environmental Resources were identified as a substitute for HCP. None of the women spontaneously received or saw any informative material such a leaflet or flyer about PA during pregnancy. Most of them looked for information on the internet, books, or TV shows to find answers to their questions. These resources answered some of their questions. However, for some of them, this information did not reassure them that moderate-to-vigorous PA was safe, because they were not sure if the source was reliable enough.

*Behavioral determinants based on the Theoretical Domains Framework (TDF) [41] mapped into the Capability-Opportunity-Motivation-Behavior (COM-B) model [37]. *HCP* Healthcare professionals, mostly obstetricians, *PA* Physical activity

**Table 4:** How incomplete professional communication shapes women’s behavioral determinants and decisions about physical activity during pregnancy

Behavioral determinants* (TDF-COM-B)	Barriers	Examples of quote	Summary
Knowledge - Psychological Capability	- Lack of knowledge was systematically cited as a barrier. - The absence or incomplete information received from HCP negatively influenced the decision-making process.	*I was expecting more suggestions like. Related to my situation [gestational diabetes]*,* what activities they recommend and how many times a day*,* for example*,* but I always get a very general answer like “Oh yes*,* of course you can exercise*,* but not the vigorous activities”.* (Pregnant woman 2, nulliparous, second trimester, maternity hospital) *With my obstetrician*,* I had to be proactive and ask questions. And then it’s always a bit like*,* “It’s as you feel. You shouldn’t jump with a parachute” [.]. I’m not particularly instinctive*,* so it’s complicated. [.] And then*,* my midwife*,* no*,* we haven’t really talked about it.* (Pregnant woman 1, primiparous, second trimester, independent obstetrician, and midwife)	All women reported that HPC did not present the recommendations and benefits of PA. Even being (partially) active before pregnancy, none of them knew the PA recommendations during pregnancy. Most pregnant participants guessed the benefits of PA with common sense based on their personal experience. They were not aware of the benefits for their child. All of them expressed regrets not having more information from HCP. They were interested in knowing the health benefits of PA for themselves and their child, as well as which type, intensity, frequency, and duration of PA were recommended during pregnancy.
Beliefs about Consequences - Reflective Motivation	- Beliefs about Consequences were mostly cited as a barrier, linking with lack of knowledge. - The beliefs of harmful consequences of PA negatively influenced the decision-making process.	*I know I saw that you shouldn’t be breathless for the baby. And I think running isn’t good for the perineum. And I don’t think riding is good either.* (Pregnant woman 9, nulliparous, third trimester, independent obstetrician and midwife) *I don’t know about that. That I don’t know. No matter how hard I look for information*,* nobody gives me a straight answer. Yes*,* I can imagine that in any case during the first trimester*,* with the shock of running for example*,* it could have an impact on the cervix.* (Pregnant woman 1, primiparous, second trimester, independent obstetrician)	Most pregnant participants reported that incomplete and contradictory information led to a confused understanding of PA consequences. They all assumed that PA is beneficial for them as they had experienced the benefits of PA based on their previous experiences. However, some women wondered if PA is as important, or even deleterious during pregnancy considering that HCP do not spontaneously address the topic. Most of them believed that moderate-to-vigorous intensity PA could be harmful for the baby and some for their perineum.
Emotion - Automatic Motivation	- Emotions were cited as a barrier in regard of beliefs about harmful consequences. - Negative emotional experiences negatively influenced the decision-making process.	*She told me I could carry on and then*,* OK*,* that was it. We didn’t discuss at all whether physical activity was good or not. And then during the pregnancy*,* we never talked about it again. [.]I had an ectopic pregnancy before my first pregnancy. [.] I think the fear was very much linked to that episode.* (Pregnant women 4, primiparous, third trimester, independent obstetrician, and midwife) *My pregnancy had an impact on how I feel about what I can do. So*,* there is the feeling of competence*,* but there’s also fearfulness. The fear of doing things wrong. So that is what really influenced what I continued to do.* (Pregnant woman 12, nulliparous, third trimester, independent obstetrician and midwife)	All women reported concerns about PA linked to the fear of harming their child. Women reported that incomplete and contradictory information led to fears and anxieties. Negative emotions were associated with past complications such as medically assisted procreation, miscarriages, ectopic pregnancies, or alarmist speeches about sport from their entourage (relatives, friends and/or colleagues), or the absence of clear answer from HCP about medical condition and type of PA.

*Behavioral determinants based on the Theoretical Domains Framework (TDF) [41] mapped into the Capability-Opportunity-Motivation-Behavior (COM-B) model [37]. *HCP* healthcare professionals, mostly obstetricians, *PA* Physical activity

Women’s behavioral determinants in regard of professional practice interacted through two overarching mechanisms: a “missed opportunity for evidence‑based information” and a “misinformation cascade” due to lack of spontaneous and accurate information in care. Together, these mechanisms constrained informed decision‑making about engagement in PA during pregnancy.

Women reported that PA information depended largely on their own initiative, as healthcare professionals, mostly obstetricians, rarely raised the topic unprompted and often provided incomplete or generic guidance. When PA was discussed, information typically focused on maintaining gently previous habits without assessing PA levels, beliefs, or needs, leaving women, especially those engaging in moderate-to-vigorous-intensity PA, unsatisfied and uncertain. Many therefore searched for information independently to determine which activities were safe. External resources were often viewed as unreliable, leading many women to reduce or discontinue moderate‑to‑vigorous PA.

The “missed opportunity for evidence‑based information” illustrated how women’s characteristics, initiative, and circumstances influence their access to PA information (Table 3). This theme drawn on two TDF domains : Social Influences and Environmental Context and Resources, mapped into Social and Physical Opportunity determinants of the COM‑B model [41].

The “misinformation cascade” illustrated how inaccurate and inconsistent information from healthcare professionals, mostly obstetrician, generated misbeliefs about PA, leading to uncertainty and fear (Table 4). This theme drawn on three TDF domains: Knowledge, Beliefs about Consequences, and Emotion, mapped into Psychological Capability, Reflective Motivation, and Automatic Motivation determinants of the COM‑B model [41].

### Qualitative results – midwives’ perspective

#### Midwifery participant characteristics

Nineteen midwives participated in semi-structured interviews. Eight were recruited at CHUV and 11 at RHNe. Most midwives reported participating in the study because they perceived a lack of skills related to PA communication and frequently experienced discomfort when addressing this topic with women. They expressed interest in improving their clinical practice and the quality of care.

Midwives at CHUV had between 8 and 30 years of professional experience across various units, with most having worked for at least 8 years in the Outpatient unit providing pre- and postnatal consultations. Their initial training in nursing and midwifery was completed in Italy, Belgium, or Switzerland and was equivalent to a bachelor’s degree.

Midwives at RHNe had worked in the maternity hospital from 2 months to 27 years and were involved in prenatal consultations, childbirth and parenthood preparation classes, antenatal care, and immediate postnatal hospitalization. They were trained in Switzerland or France, and all held at least a bachelor’s-level qualification.

#### Determinants influencing midwives’ communication about physical activity during pregnancy

Tables 5, 6 and 7 present the summary matrix of determinants with illustrative quotes. The narrative interpretation of analytic themes is presented below, with the full set of themes and sub‑themes provided in Additional file 4. 

**Table 5:** How women’s characteristics shape midwives’ communication on physical activity during pregnancy

Behavioral Determinants* (TDF-COM-B)	Barriers	Examples of quote	Summary
Memory, Attention, and Decision processes - Psychological Capability	- Memory, Attention, and Decision processes were identified as a barrier and associated with Behavioral Regulation. - Midwives’ one-sided willingness to address PA negatively influenced the systematic delivery of PA communication.	*If I provide information is in relation to women who give me indications of specific pain.* (Midwife 4, Hospital 1, 31 years’ experience) *I don’t think about it. It’s just that it’s not part of what I’m used to saying.* (Midwife 14, Hospital 2 10 years’ experience)	Midwives decided when to talk about it in a non-systematic way. They addressed the topic in accordance with some pregnancy-related conditions such as back pain, constipation, excessive weight gain or delivery. Midwives reported to not think about PA if it had no function of treatment or if women don’t speak spontaneously about it.
The Social Influences - Social Opportunity	- The Social Influences were identified as a barrier and associated with lack of training and procedure. - The profile of the pregnant women influenced communication about PA.	*Often*,* it’s not necessarily the woman who has questions to ask*,* because if she asks me questions, I’m obviously going to go further*,* but she’s not asking me any questions.* (Midwife 4, Hospital 1, 31 years’ experience) *If I see an obese woman at the end of her pregnancy at 36 weeks, I say to myself*,* it’s a bit inappropriate to tell her now “Madam*,* you need to move”. If it’s never been said before*,* then I’m going to be less*,* well*,* how can I say it? I’ll be less insistent.* (Midwife 6, Hospital 1, 17 years’ experience) *In a way*,* I don’t feel legitimate. I think they’re already pregnant*,* they’ve got a lot to deal with*,* so if on top of that I tell them to start a sport. Well*,* I have a mirror effect with myself. I tell myself that when I’ve got time*,* when I’ve got nothing to deal with*,* I don’t do it*,* so these pregnant women...* (Midwife 17, Hospital 2, 2 years’ experience)	The topic was less likely to be discussed or will be just evoked if pregnant women do not ask questions, look not interested, do not seem to need it, seems to be overloaded, do not speak the same language as the midwife, or are too thin or too overweight. PA are avoided if midwives thought that women will associate this with a constraint.

*PA* Physical activity

*Behavioral determinants based on the Theoretical Domains Framework (TDF) [41] mapped into the Capability-Opportunity-Motivation-Behavior (COM-B) model [37].

**Table 6:** Training-related key determinants influencing midwives’ communication about physical activity during pregnancy in routine care

Behavioral Determinants* (TDF-COM-B)	Barriers	Examples of quote	Summary
Skills - Psychological Capability	- Lack of Skills were systematically cited as a barrier. - Low level of Skills negatively influenced communication about PA.	*So not at school*,* clearly not. I mean*,* as far as I remember*,* we didn’t talk about physical activity and pregnancy. My colleagues taught me when I arrived*,* and I consulted with them for several months. I took on board what they were saying. And then*,* my personal experience*,* that’s it.* (Midwife 2, Hospital 1, 14 years’ experience) *Sport is very much a part of my life. I think it has a big influence. When you love something*,* you tend to talk about it positively and with envy.* (Midwife 19, Hospital 2, 2 years’ experience)	All midwives highlighted their lack of training. They developed Skills from their experiences in the field and personal life. Midwives perceived that women who were physically active during pregnancy appeared healthier. Some read books, have searched on the internet, attended conferences in their personal time, or learned by imitation from their colleagues. Recently graduated midwives (< 5 years of experience) reported receiving a 1-2-hour course on PA during pregnancy.
Knowledge - Psychological Capability	- Lack of Knowledge was systematically cited as a barrier. - Low level of Knowledge negatively influenced communication about PA.	*I can’t tell you exactly what they [recommendations] are*,* but I think that they can at least have an activity that is. They shouldn’t be inert; they shouldn’t just lie there throughout their pregnancy. On the contrary*,* they need to move around*,* they need to get some fresh air*,* they need to get active*,* and in fact there’s nothing wrong with movement.* (Midwife 1, Hospital 1, 20 years’ experience) *I usually try to use common sense. Like this morning*,* I had a couple who said “we like hiking. On Saturday*,* we went up to 2000 meters and hiked for 4 hours. Is that OK? I said “yes*,* if you’ve done it before*,* you can go gently”.* (Midwife 5, Hospital 1, 20 years’ experience) *I know it’s good to move during pregnancy. But then*,* what? how? And how much? I have no notion.* (Midwife 13, Hospital 2, 5 years’ experience)	No midwife was aware of national or international recommendations of PA during pregnancy. They described having some notions about (1) the duration and frequency of PA, e.g., 30 min per day, (2) the type of PA e.g., walking, swimming, yoga, no sports with risks of falls and trauma. However, none of them mentioned intensity or breathlessness. In most of the testimonies, there was an ambivalence between women can continue their habits and they should not do too much and/or stop sport. Midwives inferred most PA benefits based on common sense rather than physiological knowledge. Some benefits for the woman were known. Benefits for children were unspecified.
Beliefs about Capabilities - Reflective Motivation	- Beliefs about Capabilities were often cited as a barrier. - A low perception of competence negatively influenced communication about PA.	*Being more at ease*,* I guess. I think that’s the most important thing*,* because being at ease makes it easier to talk about it. And so*,* without even realizing it*,* we introduce it into our consultations. We try to avoid the subject because we don’t know how to answer anyway*,* so.* (Midwife 3, Hospital 1, 13 years’ experience) *We would have to find the right words. How to bring that to her. So*,* I’m not saying I wouldn’t be able to do it*,* but how do I get it to her without hurting her feelings?* (Midwife 10, Hospital 2, 21 years’ experience)	Their confidence in communicating PA information varied depending on perceived skill and knowledge, personal PA habits, the woman’s profile, and type of consultation. The topic is avoided if the midwife felt not at ease to address the topic. Two main reasons of avoiding the subject are: feeling they lacked sufficient knowledge to answer correctly and not knowing how to address the topic in a non-judgmental way.

*Behavioral determinants based on the Theoretical Domains Framework (TDF) [41] mapped into the Capability-Opportunity-Motivation-Behavior (COM-B) model [37]. *PA* Physical activity

**Table 7:** Resources-related key determinants influencing midwives’ communication about physical activity during pregnancy in routine care

Behavioral Determinants* (TDF-COM-B)	Barriers	Examples of quote	Summary
Behavioral Regulation - Psychological Capability	- Lack of Behavioral Regulation was frequently cited as a barrier. - Lack of procedure in the maternity hospital negatively affected the systematic delivery of PA communication.	*No*,* I don’t have a plan*,* that’s for sure. Often*,* I let myself be guided by the woman and I would say that there are patients to whom I don’t talk about it at all.* (Midwife 3, Hospital 1, 13 years’ experience) *it’s not something that’s written in big red*,* I must talk about it*,* I admit*,* it’s not.* (Midwife 16, Hospital 2, 27 years’ experience)	PA is not a mandatory topic to be addressed unlike other health behaviors such as diet and substance misuse. The midwives with complementary activities in parenting classes and/or pelvic floor consultation noted that it was easier to talk about PA in these consultations because it is part of the content of the appointment.
Environmental Context and Resources - Physical Opportunity	- Lack of Resources such as informative supports were cited as a barrier. - The absence of informative resources negatively influenced communication about PA.	*I think some kind of support would be good. So that each of us doesn’t do our own thing and come up with different recommendations. Well*,* we should all have more or less the same speech about what we can say [on PA].* (Midwife 7, Hospital 1, 8 years’ experience) *It’s very simple*,* but to have leaflets*,* maybe the first few days you think “Oh yeah*,* I’ll talk about that a bit more”. So*,* you need a visual. [.] Things to remind you that it’s good.* (Midwife 14, Hospital 2, 10 years’ experience)	There is no informative resource on PA available in the maternity hospital to support the delivery of evidence-based information. Informative resources are perceived as reliable sources of information and trigger to remember to address the topic.

*Behavioral determinants based on the Theoretical Domains Framework (TDF) [41] mapped into the Capability-Opportunity-Motivation-Behavior (COM-B) model [37]. *PA* Physical activity

Midwives’ behavioral determinants in regard of PA professional practice interacted through two overarching and limiting conditions: lack of training and lack of standardized procedures and resources. Together, these limiting conditions resulted in “lack of spontaneous delivery” and “lack of accurate and tailored information” on PA during pregnancy and made the topic invisible in maternity care.

Midwives rarely addressed PA spontaneously and generally discussed it only when women initiated the topic. Limited knowledge, low confidence, and uncertainty about recommendations and benefit led many to avoid the subject or provide vague, non-specific information. When PA was discussed, guidance typically focused on “gently” maintaining prior activity, without assessing individual PA levels, beliefs or needs. Overall, PA communication was inconsistent, and dependent on women’s questions.

The “lack of spontaneous delivery” by midwives illustrated how current communication on PA depends on women’s characteristics (Table 5). This theme drawn on two TDF domains: Social Influences and Memory, Attention, and Decision processes, mapped into Social Opportunity and Psychological Capability determinants of the COM-B model [41].

The “lack of accurate and tailored information” by midwives illustrated how communication on PA depends on environmental determinants such as training (Table 6), procedures and resources (Table 7). This theme drawn on five TDF domains: Skills, Knowledge, Beliefs about Capabilities, Behavioral Regulation, and Environmental Context and Resources, mapped into Psychological Capability, Reflective Motivation, and Physical Opportunity determinants of the COM-B model [41].

### Integration of women's and midwives’ perspectives

#### Integration of quantitative and qualitative results from women

The integration of findings from pregnant participants revealed concordant and complementary patterns (Additional file 3). Overall, results revealed that PA promotion during pregnancy was not delivered systematically and influenced by women’s characteristics. Slightly less than 40% of women reported receiving no information and higher perceived knowledge was associated with higher pre-pregnant PA level. Qualitative data indicated that discussions about PA often depended on women’s proactivity.

Women reported that healthcare professionals mostly advised to continue with their PA habits (44%) while interviewed women reported that they vaguely advised maintaining it “gently”, with limited assessment of their PA levels, beliefs or needs. Interviewed women engaged in moderate-to-vigorous PA expressed greater dissatisfaction when information was perceived as unclear, untailored, or brief.

Across data sources, a substantial proportion of women reported incomplete knowledge of PA recommendations and expressed a need for more information. Qualitative findings further suggested that many women relied on personal initiative and external resources to compensate for gaps in professional guidance.

Together, these findings highlight missed opportunities for tailored and evidence-based PA communication during pregnancy and suggest a selective pattern in PA promotion practices. Qualitative data indicated that insufficient access to clear and reliable information from professional appeared to contribute to the reduction or discontinuation of moderate-to-vigorous intensity activities during pregnancy.

#### Integration of qualitative results from midwives and integrated results from women

The integration of findings from pregnant women and midwives revealed concordant and complementary patterns (Additional file 4). Overall, both midwives and women demonstrated gaps in knowledge regarding PA recommendations and benefits. Results indicated that PA promotion practices were often inaccurate and inconsistent across public and private care.

Midwives’ accounts indicated that limited PA promotion practices were primarily due to insufficient training, lack of standardized procedures, and limited resources.

Midwives’ PA guidance was typically brief and oriented toward “gentle” PA, reflecting ambiguity about which types and intensities of PA are recommended during pregnancy and why. Consistently, women reported that healthcare professionals, mostly obstetricians, generally recommended maintaining PA “gently”, with little individual assessment.

Midwives tended to avoid the topic when they were not confident in PA knowledge or communication skills. This aligns with women reported that they often needed to initiate discussions and perceive healthcare professionals, mostly obstetricians, as insufficiently engaged or up to date on the topic.

Overall, women who actively asked about PA were more likely to receive information, suggesting that PA promotion remains largely reactive rather than routinely initiated by healthcare professionals, including midwives, despite partial awareness of its benefits.

#### Integrating multilevel determinants to inform a PA-communication framework

**Fig. 1:**
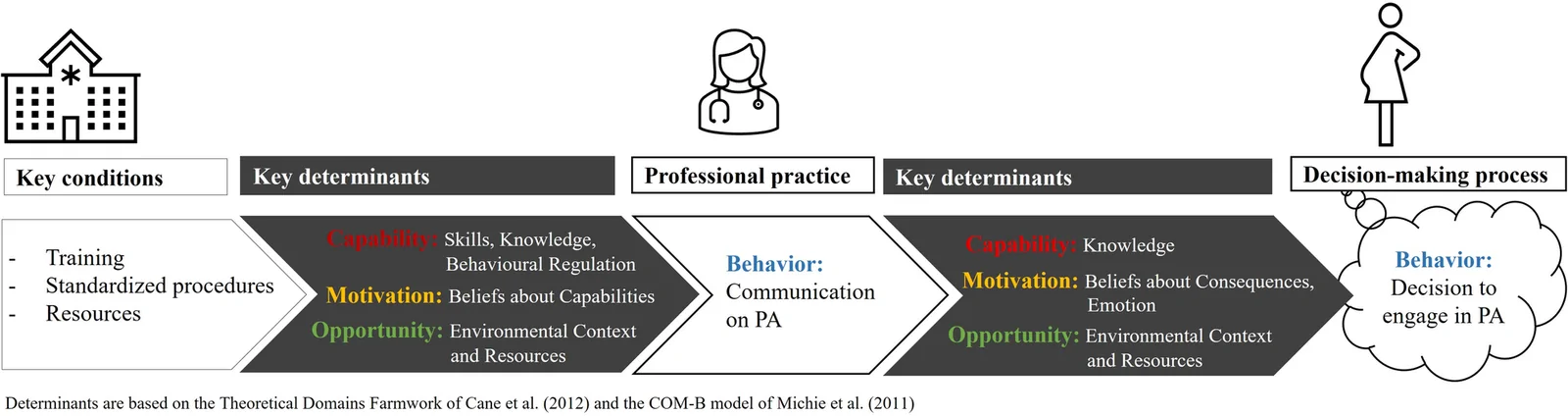
Framework of interrelated multilevel determinants shaping PA communication and decision‑making in pregnancy

Figure 1 illustrates how women’s and hospital-based midwives’ behavioral determinants converge to inform the mechanisms underlying effective PA communication in routine pregnancy care. Professional multilevel determinants, shaped by training and work conditions, interact with women’s behavioral determinants and influence decisions to engage in PA during pregnancy.

Limited training, procedures, and resources reduced hospital-based midwives’ Capability, Opportunity, and Motivation, leading to a lack of evidence‑based, tailored, and spontaneous PA information. In turn, women reported receiving inaccurate, non‑tailored, or non‑spontaneous PA communication, often from their independent obstetrician. This inaccurate communication reduced their Capability, Opportunity, and Motivation to engage in PA, particularly among those uncertain about moderate‑to‑vigorous activity, due to misbeliefs and negative emotions.

Using the COM-B, and the Behavior Change Wheel [37], strengthening professional knowledge, skills, context, and confidence through training, procedures and resources, could enhance first professional Capability, Motivation, and Opportunity. In turn, women’s Capability, Opportunity, and Motivation could be improved through more spontaneous and systematic PA communication. To be effective, PA communication should be tailored to women’s individual knowledge, beliefs, and emotions.

## Discussion

The aim of this mixed-methods study was to better understand how behavioral determinants, professional PA promotion practices, and pregnant individuals’ characteristics collectively influence decisions to engage in PA during pregnancy.

Access to PA information during prenatal care often relied on women’s own initiative or perceived interest. In our sample, over one‑third reported that PA was not addressed, and more than half expressed a need for additional guidance. The absence of evidence‑based, tailored, and spontaneous communication contributed to misbeliefs and negative emotions that shaped PA decision‑making. Inconsistencies and inaccuracies in hospital-based midwives’ communication appeared linked to limited training and the lack of standardized procedures and resources.

By examining these multilevel determinants using the TDF domains and the COM-B model, we identified multilevel mechanisms explaining why PA communication remains inconsistent in routine care. The resulting framework highlights key environmental determinants that interact with individual determinants among both professionals and women.

### Limiting environmental conditions

Overall, our results point to a systemic barrier: hospital-based midwives, and likely other healthcare professionals, lack structural and educational support to address PA confidently. Their resulting uncertainty, perceived by women, contributes to confusion about the safety of PA during pregnancy.

Training and working condition barriers have been widely reported in previous research on PA promotion during pregnancy, particularly among midwives [10, 19, 26, 27, 29]. Avoidance of the topic has also been documented when obstetricians or midwives perceive PA counselling as challenging during consultations [27, 50, 51]. Such avoidance may undermine trust in professional advice and contribute to lower intention to engage in PA during pregnancy [52].

### Access to physical activity information during pregnancy

This study highlights how non-spontaneous and inconsistent PA communication in maternity care created missed opportunities to deliver evidence-based information, ultimately hindering decision-making and reinforcing inequities in access to information.

These findings align with the international evidence indicating limited PA promotion practice in prenatal care [5, 19, 25]. For example, studies from Australia reported that approximately one-third of healthcare professionals did not raise the topic with pregnant women [23], and around half of pregnant respondents did not receive information from their medical practitioners [53]. Similarly, a recent scoping review of PA communication during pregnancy in UK highlighted that discussions about PA are often inconsistent [19].

More broadly, previous research showed PA levels during pregnancy reflects social inequalities [1, 54]. Women with lower income or education levels and those from minority backgrounds have reduced access to health information, including PA, due to lower health literacy and structural barriers within health systems [55, 56]. Environmental constraints, such as limited healthcare and community resources, including few low‑cost pregnancy‑specific programs, further restrict opportunities for PA information during pregnancy [15, 16, 18, 19].

### Accuracy of physical information during pregnancy

This study highlights how incomplete and inaccurate PA communication in maternity care fostered misbeliefs and negative emotions toward PA, ultimately hindering decision‑making and reinforcing inequities in access to information. Neither the interviewed women nor the midwives were aware of national guidelines, which have existed in Switzerland since 2018 [57].

These gaps reflect a broader structural issue: PA guidelines are poorly accessible and disseminated worldwide [58]. For example, only 2% of midwives in a British study accurately reported national guidelines despite 59% feeling confident addressing PA questions [24], and an Australian study showed that healthy pregnant women engaging in moderate‑to‑vigorous PA were often advised to reduce to mild‑intensity activity [23].

Consistent with previous research, knowledge gaps regarding PA during pregnancy persist across health professions, including midwives [5, 19, 25], with information often incomplete or insufficiently tailored [9, 19, 23, 53]. Limited knowledge and negative emotions among pregnant women are also well‑established barriers to PA engagement [13, 15, 16, 18, 19, 38].

### Practical applications

Evidence from international guidelines and meta-analyses shows that prenatal PA is safe, beneficial, and recommended [1, 2]. However, our findings and previous research indicate that PA communication remains poorly and inconsistently integrated into routine care [8, 9, 19]. This implementation gap persists despite clearly identified levers for action and reflects primarily structural rather than individual barriers [6, 8, 35]. Addressing it requires structured multilevel strategies to translate evidence into real-world care [8, 19, 39, 48]. Hospital-based midwives play a central role, particularly for families from socially disadvantaged groups, but sustained change requires consistent, evidence-based PA communication across maternity care professions [5, 6, 55].

Based on our findings and previous research, we therefore propose a multilevel-strategic framework using the Behavior Change Wheel [37, 42] to strengthen communication about PA during pregnancy by healthcare professionals, particularly hospital-based midwives. Additional file 5 provides the theoretical mapping of determinants and examples.

#### Policy, educational and professional levels

Integrating pregnancy-specific PA content into healthcare education and continuing training is essential to address knowledge, skills and confidence gaps limiting effective PA communication [9, 10, 48]. Training should cover evidence-based recommendations, benefits, safety, and practical communication strategies [19, 59, 60]. Emphasizing that PA is not an additional burden but a resource for women’s well-being, and providing experiential training exposing professionals to different types and intensities of PA, may support understanding and promotion in practice [48, 61].

#### Health system and hospital levels

User‑friendly guidelines and clinical pathways can help embed PA discussions in routine care and address time and organizational barriers commonly reported by midwives [19, 25]. In our sample, most midwives reported forgetting or not prioritizing PA. Clear procedures and prompts may therefore support more consistent communication and reduce informational disparities.

#### Resource level

Concise standardized tools, such as leaflets, infographics, digital tools, can support clear and efficient PA communication in real-word care [10, 19, 26]. Women and midwives in our study expressed the need for accessible and reliable resources from trusted institutions. Official time‑saving materials summarizing PA recommendations and benefits may help unify information.

#### Care‑relationship level

This framework calls for spontaneous, regular, tailored, and bidirectional communication about PA recommendations and benefits, adapted to women’s symptoms, needs, and preferences, reflecting their demand for personalized and trustworthy guidance [9, 14, 40, 52]. Women in our study highlighted the need for positive, evidence‑based information explaining both the “how” and the “why” of PA to counter misbeliefs and negative emotions.

### Limitations

This study has several limitations. The purposive sampling strategy without incentives for pregnant women may have introduced selection bias. Recruiting less physically active women was particularly challenging, resulting in a sample largely composed of active, highly educated participants with a strong interest in improving current practices. Consequently, some subgroups were underrepresented, reflecting a common recruitment bias whereby individuals less interested in the topic, who may perceive PA during pregnancy as risky, or who feel the study does not address their needs, are less likely to take part [1, 62].

In addition, only hospital-based midwives were interviewed. Although women reported receiving PA information from various healthcare professionals, these accounts reflect perceptions rather than direct evidence of other professionals’ practices. As a result, the identified determinants and strategies may not be transferable to all healthcare professionals and private care.

Finally, while the TDF domains are widely used to analyze behavioral determinants, evidence for its predictive capacity remains limited. To strengthen the theoretical interpretation, it was combined with the COM-B model and the Behavior Change Wheel, which support the translation of determinants into potential intervention strategies [36, 37].

### Future research

Further research should examine the interrelated determinants influencing PA communication during pregnancy across a broader range of healthcare professionals. Including more diverse populations of pregnant people, particularly those with lower PA levels or different socioeconomic backgrounds, would also help better understand varying information needs and reduce inequalities in access to PA guidance.

Future work should also test evidence-based strategies designed to improve PA communication under real-word care conditions. In this perspective, a mixed-methods interventional study is currently being conducted to implement communication strategies directly in routine maternity care and examine changes in professional practices and pregnant people’s experiences over time.

## Conclusions

This mixed-methods study examined how behavioral determinants, healthcare professionals’ PA promotion practices, and pregnant people’s characteristics shape decisions about PA during pregnancy. Findings revealed persistent gaps in the communication of PA recommendations and benefits in routine maternity care, with access to information often depending on women’s proactivity. These gaps reinforced misconceptions and negative emotions that negatively influenced PA decision-making, highlighting the need for systematic, spontaneous, and tailored communication on PA during pregnancy to foster PA engagement.

Addressing these issues requires structural and organizational changes to support hospital-based midwives and other healthcare professionals in delivering spontaneous and evidence-based information.

Future research should focus on testing and scaling theory-informed strategies within routine care to identify feasible approaches to improve PA communication. At the policy level, integrating PA guidance into professional education and maternity care standards, and embedding PA promotion within interdisciplinary care pathways, will be key to ensuring equitable access to reliable PA information for all pregnant people.

## Supplementary Information

Supplementary Material 1.

Supplementary Material 2.

Supplementary Material 3.

Supplementary Material 4.

Supplementary Material 5.

## Data Availability

The datasets used and/or analysed during the current study are available from the corresponding author on reasonable request.
