# Supplementary material for: Handling the challenges of accessing reliable and tailored information on physical activity during pregnancy: a mixed-methods, cross-perspective investigation
Source: BMC Pregnancy Childbirth. 2026 Jun 15;26:940. doi: 10.1186/s12884-026-09482-7 (PMC13501815; doi:10.1186/s12884-026-09482-7)
Supplement: Supplementary file 1 [file 12884_2026_9482_MOESM1_ESM.docx]

**Additional file 1 – quantitative and qualitative data collection tools (English version)**

*Quantitative- Items from the second part of the Survey*

**About you**

1. During your pregnancy you are mainly under the follow-up of:

□ *maternity hospital*

*□ A obstetrician-gynecologist in private practice*

*□ An independent midwife*

*□ Other….*

2. What trimester of pregnancy are you in?

*□ 1^st^ trimester*

*□ 2^nd^ trimester*

*□ 3^rd^ trimester*

*□ I don't know*

**Source of information**

3. Has any health professional (gynecologist, midwife, nurse, physiotherapist...) addressed the issue of physical activity with you at least once?

*□ Yes*

*□ No*

*□ I don't remember*

3.1. If yes, they recommended that you

*□ maintain your physical activity*

*□ reduce your physical activity*

*□ increase your physical activity*

*□ other….*

4. Do you have any complications related to your pregnancy? (multiple choice)

*□ None*

*□ Joint pain (back, pelvis...)*

*□ Fluid retention*

*□ Varicose veins*

*□ Hypertension*

*□ Gestational diabetes*

*□ Anemia*

*□ Excessive weight gain*

*□ low placenta*

*□ Other, specify...*

4.1. If you have one or more complications, have health professionals addressed with you the issue of physical activity in relation to the complication(s)?

*□ Yes*

*□ No*

*□ I don't remember*

5. For each of the following statements, please indicate your level of agreement or disagreement

|  | *Strongly agree* | *Agree* | *Neither agree nor disagree* | *Disagree* | *Strongly*  *disagree* |
| --- | --- | --- | --- | --- | --- |
| *I am satisfied with the information I received from health professionals about physical activity.* | *❑* | *❑* | *❑* | *❑* | *❑* |
| *I am satisfied with the responses I received from health professionals to my questions about physical activity.* | *❑* | *❑* | *❑* | *❑* | *❑* |
| *I would have appreciated more information from health professionals about physical activity during and after pregnancy.* | *❑* | *❑* | *❑* | *❑* | *❑* |

**Knowledge on physical activity**

6. Are you familiar with physical activity recommendations during and after pregnancy?

□ Yes

□ Rather yes

□ Rather no

□ No

□ No opinion

**View on physical activity**

7. Before pregnancy you considered yourself to be

*□ Athletic, very active (you played a sport regularly and more than 2.5 hours per week)*

*□ Physically active (you moved around in daily life, limited sitting time, and engaged in physical activities (commuting, stairs, yoga, dance, walking, biking...) that made you slightly out of breath at least 2.5 hours per week)*

*□ Partially active (you were performing physical activities that made you slightly out of breath between 30 min. and 2.5 hrs. per week and/or had sedentary activities for several hours without interruption (sitting and/or lying down with little movement).*

*□ Inactive (you performed less than 30 min. of physical activity that made you slightly out of breath and you spent long periods of time performing activities in an immobile position (sedentary activity)).*

**About you**

8. What is your age range?

*□ 18 -24 years old*

*□ 25-30 years old*

*□ 31-40 years old*

*□ over 40 years old*

9. What level of education do you have? (Indicate your highest degree)

*□ Obligatory school*

*□ Secondary level: vocational or general*

*□ Tertiary level (University)*

*□ I do not wish to answer*

10. Do you have a comment to share with us?

***…………………………………………………………***

*Qualitative-* *Interview guides for women and midwives (English versions)*

**Table S1.** Interview guide questions for women

| **Topic** | **Question** | **Prompt** |
| --- | --- | --- |
| **About you** | - How is your pregnancy going in general? | - What stage are you at in your pregnancy/ post pregnancy? - Is this your first pregnancy? |
| **View on physical activity** | - What does physical activity evoke in you? | - In general, during pregnancy or post-partum? |
|  | - How do you move in your everyday and leisure life? | - Are there any differences between before and during/after pregnancy? - What motivates you to be physically active? - What hinders you from being physically active? - Do you feel able to do regular physical activity? |
| **Source of information** | - How have you been informed about physical activity during and/or after pregnancy? | - Has the medical profession talked to you about physical activity? - If so, how and what? How did you feel during this discussion? - If not, would you have liked them to talk about it? How? - Did you ask any questions about it? - Were you satisfied with the information received? If not, what did you need? |
|  | - Have you talked about physical activity with other people? | - If so, with whom? What was the content? - How did you feel about this/these discussion(s)? |
| **Knowledge on physical activity** | - What do you think are the recommendations for physical activity during/after pregnancy? | - In your opinion, what are the benefits of physical activity? - And for your baby? - Do you think that physical activity could be dangerous for you or your baby? Why? |

**Table S2.** Interview guide questions for midwives

| **Topic** | **Question** | **Prompt** |
| --- | --- | --- |
| **About you** | - How long have you worked in the maternity unit? | - What is your activities? |
| **View on physical activity** | - What does physical activity evoke in you? | - In general, during pregnancy or post-partum? |
| **Practice of physical activity promotion (source of information)** | - How and when do you talk to women about physical activity? | - Do you use resources to talk about physical activity? - What health behaviours do you discuss with women? During which visits? - What are the barriers to talking about physical activity? - What enables you to talk about physical activity? |
|  | - How do you feel when you talk about physical activity? - What is your role in promoting physical activity in perinatal care? |  |
| **Knowledge on physical activity** | - How would you define physical activity | - What do you think are the recommendations for physical activity during/after pregnancy? - In your opinion, what are the benefits of physical activity? For the women, for the baby? - Do you think that physical activity could be dangerous the women or the baby? Why? |
|  | How did you obtain this information? | - How do you move in your everyday and leisure life? |
