# Supplementary material for: Handling the challenges of accessing reliable and tailored information on physical activity during pregnancy: a mixed-methods, cross-perspective investigation
Source: BMC Pregnancy Childbirth. 2026 Jun 15;26:940. doi: 10.1186/s12884-026-09482-7 (PMC13501815; doi:10.1186/s12884-026-09482-7)
Supplement: Supplementary file 2 [file 12884_2026_9482_MOESM2_ESM.docx]

**Additional file 2 – Coding index of qualitative analysis**

Presentation of the coding three on behavioral determinants which influence physical activity (PA) behavior during pregnancy and PA promotion practice of healthcare professionals (HCP).

**Table S3.** Coding Index on PA promotion practice and PA behavior during pregnancy based on the Theoretical Domains Framework (TDF).

| **Domain of TDF** | **Sub-themes identified from midwives’ interviews** | **Sub-themes identified from pregnant women’s interviews** |
| --- | --- | --- |
| **Knowledge**  To what extent does the person have knowledge about the behavior. | 1. Knowledge that PA could be advised during pregnancy. 2. Unawareness of Swiss or international recommendations 3. Lack of knowledge on PA recommendations and the FITT (frequency, intensity, time and type of PA) principle 4. Lack of knowledge on benefits for women and children. 5. Lack of knowledge on physiological processes 6. Lack of knowledge on healthy conversation skills 7. Knowledge of some adapted physical activity offers for pregnant women. | 1. Knowledge that PA could be advised during pregnancy. 2. Unawareness of Swiss or international recommendations 3. Lack of knowledge on PA recommendations and the FITT (frequency, intensity, time and type of PA) principle) 4. Lack of knowledge on benefits for women and children. |
| **Skills**  To what extent does the person acquire proficiency through practice to perform the behavior. | 1. (Absence of) training on PA or PA promotion 2. Obtention of information from the field by experience with women or by imitation of their colleague. 3. PA level of midwives | 1. Pre-pregnancy PA level of women 2. Physical discomfort 3. Number of pregnancies |
| **Memory, Attention and Decision processes**  To what extent does the person decide to perform the behavior. | 1. Medical condition. 2. Stage of pregnancies. | 1. Pre-pregnancy type of PA 2. Information received from HCP. |
| **Behavioral Regulation**  To what extent are there routines in place to perform the behavior. | 1. No habit 2. Optional topic in consultation 3. Routine acts for medical check-ups. | 1. Pre-pregnancy PA routine |
| **Social influences**  To what extent do other individual influence to engage or disengage in the behavior. | 1. Questions from women. 2. Complaint about discomfort come from the women. 3. Weight of women 4. Socio-economic level of women | 1. Advice from HCP 2. Advice from family 3. Advice from colleagues 4. Advised from female friends 5. Support from partner 6. Mediatization of pregnant female athletes |
| **Environmental Context and Resources**  To what extent does the perceived environmental context support the person in performing the behavior. | 1. Absence of informative material support for women 2. Absence of directive in the hospital 3. Co-consultation with obstetrician 4. Non-official website on the Internet 5. Private or public health offers for PA during pregnancy | 1. Absence of informative material 2. Internet, books, or TV shows 3. Private prenatal pilates and yoga classes |
| **Social/Professional Role & Identity**  To what extent does the person feel it their responsibilities to perform the behavior. | 1. Identification of professional role of PA promotion 2. Every HCP should talk about PA 3. Self-perception of their own PA level (being sporty or not) 4. Self-experience on PA during their pregnancy | 1. Self-perception of their own PA level (being sporty or not) 2. Role of being a good mother (to protect child, spending time in family) 3. Time to take care of self 4. Management of their own pregnancy, being proactive |
| **Beliefs about capabilities**  To what extent does the person feel confident to perform the behavior. | 1. Feeling able to inform about PA recommendations 2. Feeling able to investigate PA habits 3. Feeling able to speak about changing PA habits 4. Avoid the subject of PA | 1. Feeling able to continue PA in daily life 2. Feeling able to continue moderate to vigorous PA activity, sport 3. Feeling able to increase PA |
| **Beliefs about consequences**  To what extent the person is in favour of performing the behavior in accordance of positive or negative behavior beliefs. | 1. Awareness of benefit for women 2. Awareness of benefit for the baby 3. Unawareness of the dose-response curve of PA benefit 4. Belief about negative impact of PA 5. Belief about how the women could feel if they speak about PA. | 1. Awareness of benefit for women 2. Awareness of benefit for the baby 3. Belief about negative impact of PA |
| **Emotion**  To what extent do positive or negative emotions influence engagement or disengagement in the behavior. | 1. Fear that women feel bad, guilty to be inactive | 1. Having pleasure doing PA 2. Fear of harming their child 3. Anxiety of something bad happened 4. Joy of a physiological pregnancy 5. Negative or positive emotions toward their body |
| **Intention**  To what extent does the person intend to perform the behavior. | 1. General intention to address PA 2. Fluctuation of priority to inform on PA | 1. General intention to be physically active 2. Fluctuation of intention to be sporty during pregnancy 3. Fluctuation of intention to move regarding pregnancy-related condition |
| **Reinforcement**  To what extent an element in the immediate environment does increase the likelihood of performing the behavior. | 1. Absence of item in software of hospital | 1. HCP's lack of spontaneous communication on PA topic. |
| **Optimism**  To what extent is the person confident of a positive outcome while engaging in the behavior. | 1. Feeling that it's too late for some women to talk about AP | 2. Resignation to stop sport and doing gentle activities 3. Optimistic if could carry habits as before pregnancy |
| **Goals**  To what extent the person has a mental representation of the behavior that should be perform. | 1. Lack of representation on procedure to speak about PA or recommendations | 1. Lack of representation on PA recommendations during pregnancy |
