# Supplementary material for: Handling the challenges of accessing reliable and tailored information on physical activity during pregnancy: a mixed-methods, cross-perspective investigation
Source: BMC Pregnancy Childbirth. 2026 Jun 15;26:940. doi: 10.1186/s12884-026-09482-7 (PMC13501815; doi:10.1186/s12884-026-09482-7)
Supplement: Supplementary file 3 [file 12884_2026_9482_MOESM3_ESM.docx]

**Additional file 3 – Pillar building process for women perceptions**

Presentation of the stage 4 of the Pillar Building process between questionnaire and qualitative finding among interviewed women.

**Table S4.** Integration of quantitative and qualitative results concerning women's perceptions of PA promotion practice.

| **QUANT**  Statistics | **QUANT**  Categories  (result interpretation) | **Pillar building**  Integrated themes | **QUAL**  Categories (selected **themes**/ sub-themes) | **QUAL**  Quote |
| --- | --- | --- | --- | --- |
|  |  |  | **Missed opportunity for evidence-based information** |  |
| Slightly less than 40% did not received any information about PA. | No systematic information. | **Confirmation**  PA was inconsistently addressed during consultations  **Expansion**  PA was more likely to be discussed when women expressed interest. | Information received depended on women’s characteristics. | *“With my obstetrician, I had to be proactive and ask questions”.* |
| ¾ reported having incomplete knowledge. | PA recommendations are partially known. | **Expansion**,  PA recommendations and benefits were unknown because HCP did not or partially address the topic. | Lack of knowledge on PA recommendations and benefits due to lack of evidence-based information from HCP. | *“I don't know about that. That I don't know. No matter how hard I look for information, nobody gives me a straight answer.”*  *“I know I saw that you shouldn't be breathless for the baby.”* |
| Association between reported PA level before pregnancy and reported knowledge of PA during pregnancy | PA knowledge come from personal experience | **Expansion**, Knowledge on PA is based on personal experience and gained by environmental resources. | Seek out information for themselves about habits and needs | *“But it was more me than my obstetrician”* |
| 60% wished to receive more information. | Received incomplete information. | **Expansion**,  Incomplete information provided, even when requested, with perceived discomfort from HCP | Perceived avoidance of PA promotion by HCP | *“When it came to physical activity, there was no clear recommendation. It was never precise. They just didn't really seem to know and let you try it.”* |
|  |  |  | **Misinformation cascade** |  |
| Almost half of them was told to maintain PA level | If information received, maintain habits is advised. | **Expansion**,  HCP vaguely advised to maintain PA without investigation on PA level and beliefs. | Advice focused on maintaining habits without exploring PA levels or beliefs | *“She told me I could carry on and then, OK, that was it”.* |
| Less than half felt satisfied about received information. | Heterogeneity about satisfaction of HCP practice. | **Expansion**,  Women who had moderate-to-vigorous PA are dissatisfied if they received unclear, untailored, or brief information. | Dissatisfaction with unclear, non-personalized or brief information when practicing moderate-to-vigorous PA | *“There was no clear recommendation. It was never precise. They just didn't really seem to know and let you try it.”*  *“It's as you feel. You shouldn't jump with a parachute" [...]. I’m not particularly instinctive, so it's complicated.”* |
| Association between addressing PA with HCP and the desire for more information | Subgroup of women want more information if PA was addressed. | **Expansion,**  Women who are interested about PA wanted to have more information. | Interested women expressed a desire for more information. | *“We didn't discuss at all whether physical activity was good or not.”* |
| Half of women with pregnancy-related complications discussed PA in relation to this issue. | PA could be addressed related to a pregnancy-related complication. | **Confirmation**,  PA could be partially addressed if purpose was treatment. | General information is provided if purpose was treatment. | *“I was expecting more suggestions like... Related to my situation [gestational diabetes].”* |
