# Supplementary material for: Handling the challenges of accessing reliable and tailored information on physical activity during pregnancy: a mixed-methods, cross-perspective investigation
Source: BMC Pregnancy Childbirth. 2026 Jun 15;26:940. doi: 10.1186/s12884-026-09482-7 (PMC13501815; doi:10.1186/s12884-026-09482-7)
Supplement: Supplementary file 4 [file 12884_2026_9482_MOESM4_ESM.docx]

**Additional file 4 – Pillar building process for communication on physical activity**

Presentation of the stage 4 of the Pillar Building process between women perception of PA promotion practice and qualitative findings among interviewed midwives.

**Table S5.** Integration of Pillar themes of women and qualitative results concerning midwives.

| **Women** |  | **Midwives** |  |
| --- | --- | --- | --- |
| **Integrated Pillar themes** | **Pillar building**  Integrated themes | **QUAL**  Categories (selected **themes**/ sub-themes) | **QUAL**  Quote |
|  |  | **Lack of spontaneous delivery** |  |
| PA was inconsistently addressed during consultations | **Confirmation**,  PA was inconsistently addressed, and women interested in PA were more likely to receive information from HCP. | Not used to speaking about it systematically  Absence of structured or planned approach  Low prioritization of the topic in clinical routines  Reluctance to address the topic if perceived as inappropriate | *“I don't think about it. It's just that it's not part of what I'm used to saying.”*  *“No, I don't have a plan, that's for sure. Often, I let myself be guided by the woman and I would say that there are patients to whom I don't talk about it at all”*  *“it's not something that's written in big red, I must talk about it, I admit, it's not.”*  *“I say to myself, it's a bit inappropriate to tell her now "Madam, you need to move".”* |
| Incomplete information provided, even when requested, with perceived discomfort from HCP | **Expansion**,  HCP who felt not at ease with PA promotion practice, bypassed the topic, as perceived as not mandatory | Avoidance of PA topic | *“We try to avoid the subject because we don't know how to answer anyway, so...”* |
| PA was more likely to be discussed when women expressed interest. | **Expansion**,  Access to PA information during pregnancy was largely driven by women’s own characteristics (previous habits, interest, and proactivity in seeking information). | Midwives addressed most often PA if woman asked question | *“If she asks me questions... I'm obviously going to go further, but she's not asking me any questions.”* |
|  |  | **Lack of accurate and tailored information** |  |
| HCP vaguely advised to maintain PA without investigation on PA level and beliefs. | **Confirmation,**  HCP vaguely advised to maintain PA if gentle without giving other information or investigating PA level and beliefs. | Advice to maintain PA if gently practice without further investigation. | *I had a couple who said "we like hiking. On Saturday, we went up to 2000 meters and hiked for 4 hours. Is that OK? I said "yes, if you've done it before, you can go gently.”* |
| Women who had moderate-to-vigorous PA are dissatisfied if they received unclear, untailored, or brief information. | **Expansion**,  The lack of information on type, intensity, frequency and duration received from HCP led to dissatisfaction among women who engage in PA leisure-time | Uncertainty about what type, frequency, duration and intensity of PA pregnant women could practice. | *“I know it's good to move during pregnancy. But then, what? how? And how much? I have no notion.”* |
| Women who are interested about PA wanted to have more information. | **Expansion**,  Women interested in moderate-to-vigorous activities seek more information because HCP often evade the topic due to uncertainty. | Partial responses and topic shifting due to lack of knowledge about moderate‑to‑vigorous PA. | *“Being more at ease, I guess. I think that's the most important thing, because being at ease makes it easier to talk about it.”* |
| PA could be partially addressed if purpose was treatment. | **Confirmation**,  PA could be partially addressed if purpose was treatment. | Use of PA for some pregnancy‑related conditions. | *“If I provide information is in relation to women who give me indications of specific pain.”* |
| PA recommendations and benefits were unknown because HCP did not or partially address the topic. | **Confirmation**,  PA recommendations and benefits were unknown in community and health profession. | Partially awareness of PA recommendations and benefits. | *“I can't tell you exactly what they [recommendations] are, but I think that they can at least have an activity that is... It shouldn't be inert.”* |
