# Supplementary material for: Handling the challenges of accessing reliable and tailored information on physical activity during pregnancy: a mixed-methods, cross-perspective investigation
Source: BMC Pregnancy Childbirth. 2026 Jun 15;26:940. doi: 10.1186/s12884-026-09482-7 (PMC13501815; doi:10.1186/s12884-026-09482-7)
Supplement: Supplementary file 5 [file 12884_2026_9482_MOESM5_ESM.docx]

**Additional file 5 –actionable strategies for communication on physical activity**

Tables S6 and S7 present the theoretical mapping of identified determinants, which underpins the actionable strategies proposed in Table S8.

**Table S6.** Mapping of identified determinants of prenatal PA communication to TDF domains, COM-B components and intervention functions of Behavior Change Wheel (BCW)

| **Determinant identified** | **TDF domain** | **COM-B component** | **Intervention functions (BCW)** | **Examples of Behavior Change Techniques (BCTTv1)** |
| --- | --- | --- | --- | --- |
| Lack of knowledge about PA recommendations and benefits | Knowledge | Psychological Capability | Education  Training | 5.1 Information about health consequences  4.1 Instruction on how to perform the Behavior; |
| Low confidence to discuss PA | Beliefs about capabilities | Reflective Motivation | Persuasion  Training | 15.1 Verbal persuasion about capability  6.1 Demonstration of Behavior |
| Forgetting to discuss PA | Memory, attention and decision processes | Psychological Capability | Environmental restructuring  Enablement | 7.1 Prompts/cues  12.5 Adding objects to the environment |
| Lack of time during consultations | Environmental context and resources | Physical Opportunity | Environmental restructuring | 12.5 Adding objects to the environment |
| Perceived lack of legitimacy | Beliefs about capabilities | Reflective Motivation | Persuasion  Education | 13.2 Framing/reframing; 5.1 Information about health consequences |

**Table 7.** Mapping of determinants of prenatal PA engagement among pregnant women in regard of PA communication in routine maternity care to TDF domains, COM-B components and intervention functions of Behavior Change Wheel (BCW)

| **Determinant identified** | **TDF domain** | **COM-B component** | **Intervention functions** | **Examples of Behavior Change Techniques (BCTTv1)** |
| --- | --- | --- | --- | --- |
| Limited knowledge of PA recommendations and benefits during pregnancy | Knowledge | Psychological Capability | Education  Training | 5.1 Information about health consequences  4.1 Instruction on how to perform Behavior |
| Misconceptions about risks of PA | Beliefs about consequences | Reflective Motivation | Persuasion  Education | 13.2 Framing/reframing  5.1 Information about health consequences |
| Fear of harming the baby | Emotion / Beliefs about consequences | Automatic / Reflective Motivation | Persuasion  Education | 11.2 Reduce negative emotions  5.1 Information about health consequences |
| Lack of tailored information from healthcare professionals | Environmental context and resources | Social Opportunity | Enablement, Environmental restructuring | 3.1 Social support (unspecified)  7.1 Prompts/cues |
| Lack of reliable resources | Environmental context and resources | Physical Opportunity | Environmental restructuring | 12.5 Adding objects to the environment |

**Table S8.** Actionable strategies to enhance physical activity (PA) communication during pregnancy for healthcare professionals, with a focus on hospital-based midwives based on Behavior Change Wheel and Behavior change techniques (BCTTv1).

| **Level of action** | **Actionable strategies (communication-focused)** | **Purpose / Expected impact** | **Examples** |
| --- | --- | --- | --- |
| **Policy/ educational levels** | Provide initial training on pregnancy‑specific PA recommendations and benefits for all health professionals, including midwives | Increase consistency; reduce variability; support spontaneous and repeated PA communication | Mandatory PA modules in midwifery, nursing, physiotherapy and medical curricula; assessment of PA communication competencies |
| **Policy/ professional levels** (hospital / professional bodies) | Provide regular continuing training and institutional support for PA communication | Increase consistency; embed PA communication in routine care; support spontaneous PA discussions | PA refresher courses/training; standardized PA checklist; inclusion of PA in clinical pathways |
| **Training content** (initial and continuous) | Provide training on evidence‑based PA recommendations and benefits, communication skills, standardized communication protocols, and access to informational tools and resources  In-person, online and/or experiential learning | Improve accuracy of PA messages; increase professionals’ confidence; harmonize practices | Theory; PA communication role‑plays; list of local PA opportunities; familiarization with standardized protocols and PA prompts in records; experiencing PA and pregnancy‑adapted PA class |
| **Health system/ Hospital levels** | Develop user‑friendly guidelines and ensure wide dissemination, easy access for professionals and integration of PA prompts | Facilitate adoption; sustain organizational support for PA communication | National / internal guidelines on PA; posters, infographics and leaflets in waiting rooms/ consultation rooms; intranet resources; electronic prompts in records. |
| **Resource level** | Provide concise, standardized, time-saving communication materials on PA recommendations and benefits | Increase efficiency; reduce time burden; improve clarity and recall | Infographics; leaflets; QR codes to PA videos or apps; brief communications scripts; list of free-of-charge PA classes |
| **Care relationship** | Routinely discuss PA; provide tailored information on recommendations and benefits; address misconceptions and fears; link PA to common pregnancy symptoms; emphasize adaptable and enjoyable PA | Women: improve knowledge; reduce uncertainty and fear; support shared decision‑making | Asking permission to discuss PA; asking what the woman does and likes; linking information to current symptoms (e.g. back pain, fatigue); simple, non‑technical explanations; asking about partner/family support; deciding together on next steps |
